# Yamaguchi Syndrome: A Case of Apical Hypertrophic Cardiomyopathy in a Patient From West Africa

**DOI:** 10.7759/cureus.103543

**Published:** 2026-02-13

**Authors:** Andrew S Dzebu, David Awini

**Affiliations:** 1 Cardiology, Cardiothoracic Centre, Ho Teaching Hospital, Ho, GHA; 2 Emergency Medicine, LuccaHealth Medical Specialty Center, Accra, GHA

**Keywords:** apical hypertrophic obstructive cardiomyopathy, blueberry-on-top pattern, hypertrophic cardiomyopathy, strain imaging, yamaguchi cardiomyopathy

## Abstract

Hypertrophic cardiomyopathy is the most common genetic cardiomyopathy. The apical variant, also known as Yamaguchi syndrome, is a rare condition that is reportedly more prevalent in Asian populations. It is characterized by hypertrophy predominantly limited to the left ventricular apex, with a classic "ace of spades" shaped left ventricular cavity. We present a 61-year-old male of West African descent with a chest pain syndrome and deep inverted precordial T-waves on 12-lead electrocardiography. Two-dimensional echocardiography demonstrated focal left ventricular hypertrophy limited to the apex, and speckle-tracking imaging showed reduced apical strain, findings consistent with apical hypertrophic cardiomyopathy (HCM). Risk assessment for sudden cardiac death was low. A previous invasive coronary artery angiography reported normal epicardial coronary arteries. The patient was started on carvedilol 6.25 mg orally twice daily, with resolution of the chest pain and, so far, a generally optimal quality of life after four months of follow-up.

## Introduction

Hypertrophic cardiomyopathy is the most common genetic cardiomyopathy, with a genotype prevalence of approximately 1:250-500, although it exhibits reduced penetrance and variable expressivity [1]. The apical variant, also known as Yamaguchi syndrome, is a rare condition that is reportedly more prevalent in Asian populations. It is characterized by hypertrophy predominantly limited to the left ventricular apex, with a classic "ace of spades" shaped left ventricular cavity [2]. Data on hypertrophic cardiomyopathy (HCM) in Ghana and the broader West African subregion are scanty, with no cases of apical hypertrophic cardiomyopathy reported in West Africa. We present a case of a 61-year-old male with a chest pain syndrome, with deep inverted precordial T-waves on electrocardiography, and typical echocardiographic and strain findings associated with apical HCM.

## Case presentation

We present a 61-year-old man of West African descent with a personal history of optimally controlled essential hypertension who complained of intermittent oppressive precordial pain for about four years. The pain occurs mostly at rest, lasts several minutes, and resolves spontaneously. The pain was unrelated to physical exertion. He denied dyspnea, palpitations, lightheadedness, and loss of consciousness. Physical examination was grossly unremarkable, except for a body mass index of 32 kg/m². Blood pressure was 121/79 mmHg.

**Figure 1:**
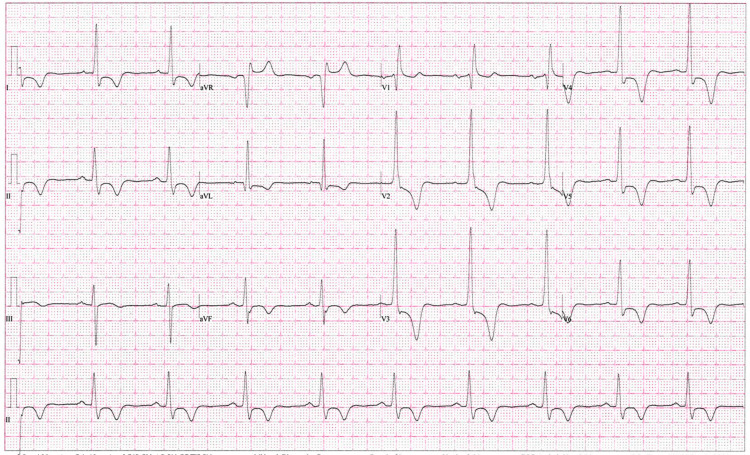
A 12-lead electrocardiogram showing ST-segment depression and deep inverted T-waves, especially in the precordial leads.

A 12-lead electrocardiography showed a sinus rhythm of about 60 beats per minute. The left ventricular voltage was normal as determined using several known methods, including Sokolow-Lyon, Cornell, and modified Cornell. There was ST-segment depression and deep T-wave inversion in the anterior leads. Corrected QT interval (using the Bazett formula) was 469 ms (Figure 1).

**Figure 2:**
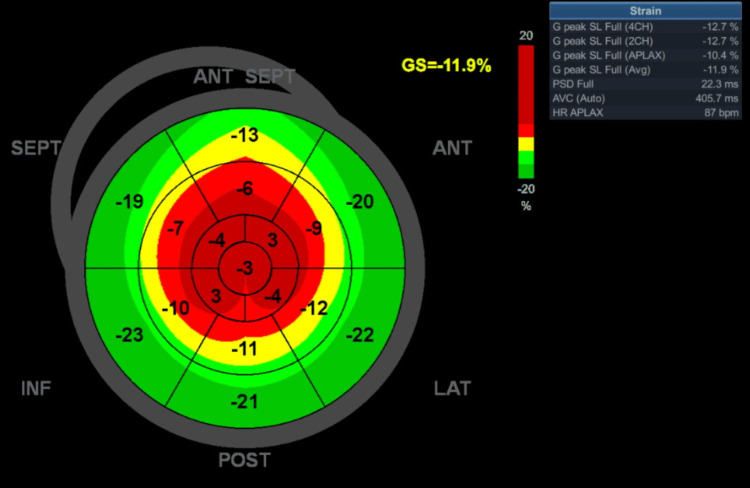
A polar map generated from strain analysis showing the loss of longitudinal strain at the left ventricular apex, a marker of myocardial disarray. ANT: anterior; SEPT: septal; LAT: lateral; POST: posterior; INF: inferior; GS: global strain; SL: segmental longitudinal strain; CH: chamber; APLAX: apical long-axis; PSD: peak systolic dispersion; AVC: aortic valve closure; HR: heart rate

Transthoracic echocardiography at rest was significant for a left ventricle with normal basal thickness (9 mm), and relatively thicker apical segments (15 mm), with cavity obliteration in those segments in systole. Global systolic function based on left ventricular (LV) ejection fraction was supranormal (80%). LV outflow tract obstruction was not present at rest or with provocative physical maneuvers. Observe the structural findings, including the classic "ace-of-spades" (Video 1). The left atrium was moderately dilated (left atrial volume index 45 mL/m^2^). Left ventricular strain analysis (performed using a GE Vivid T9 V204 Ultrasound system, with Automatic Functional Imaging version 3.0 {Chicago, IL: GE HealthCare}) showed reduced global strain (-11.9%) and a notable reduction in the apical segments (Figure 2). Cardiac magnetic resonance imaging was not pursued due to temporary challenges in obtaining access.

**Video 1:** Apical cineloops of the transthoracic echocardiography showing the morphological features of apical HCM, including the "ace-of-spades" shape of the left ventricular cavity and cavity obliteration at the apex. HCM: hypertrophic cardiomyopathy

A review of the patient’s records showed a fairly recent invasive coronary angiography, which was unremarkable. He recalls that while he was having a preoperative assessment for goiter four years ago, an abnormal electrocardiogram triggered an invasive coronary angiography. In retrospect, we hypothesize that this may have been due to the deep negative precordial T-waves, which may have been thought to represent Wellens syndrome, an ECG tracing suggestive of critical stenosis of the proximal left anterior descending artery.

The patient was further evaluated for arrhythmic features of HCM, such as non-sustained ventricular tachycardia, predictive of sudden cardiac death. Holter monitoring showed sinus rhythm only, with sustained ST-segment depression and deep inverted T-waves. A HCM Sudden Cardiac Death (SCD) Risk score of 1.07 was calculated, indicating his ineligibility for an implantable cardioverter-defibrillator. He was placed on carvedilol 6.25 mg orally twice daily to enhance lusitropy, improve myocardial perfusion, and reduce repolarization heterogeneity across the myocardium (thereby reducing the probability of arrhythmias driven by re-entry). During the first four months of follow-up, he remained asymptomatic, without recurrence of chest pain, and with normalization of the corrected QT interval.

**Figure 3:**
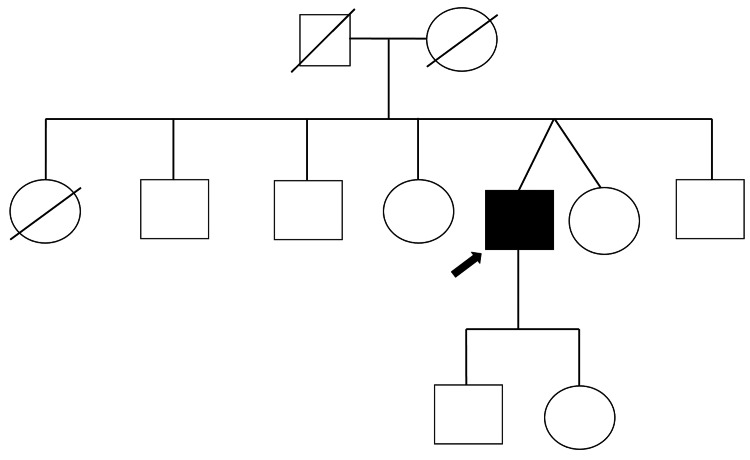
Pedigree diagram showing the proband. Square=male, circle=female, square with diagonal line=deceased male, circle with diagonal line=deceased female, black-filled square=affected male, arrow points to proband.

A family history was elicited, as required for all cases of myocardial disease. A three-generation pedigree diagram was generated (Figure 3). Based on history, no first-degree relative was reported to have died suddenly, to have symptoms compatible with HCM, or to have been diagnosed with hypertrophic cardiomyopathy. The quality of this information may be affected by recall bias. Screening was suggested for first-degree relatives, and was accepted by one, a fraternal twin, who had a 12-lead electrocardiography done, which turned out to be normal. Screening is pending for the rest of the family members.

Genetic counseling was offered to the patient. He agreed to undergo genetic screening, which is pending. It is possible that this may be a de novo mutation, as the pedigree diagram did not indicate a first-degree relative with HCM, which is common in Yamaguchi syndrome.

## Discussion

Hypertrophic cardiomyopathy is one of the most common genetic cardiomyopathies, with a genotypic prevalence of 1:250 to 1:500 [3]. It has an autosomal dominant pattern of inheritance. In the Ghanaian context, this translates to between 61,600 people (inferred from the 2020 census data). It is known to exhibit reduced penetrance and variable expression. This means that although all generations in an affected family may inherit the gene responsible for this disease, not every individual will manifest the disease phenotypically. And those who develop the disease may do so with varying degrees of severity. In clinically diagnosed HCM, mutations are identified in approximately one-third of all patients, highlighting the genetic heterogeneity and a knowledge gap that may be elucidated through further research [3].

Gene mutations are the initiating insult in the pathogenesis of HCM, affecting proteins that play a crucial role in the function of the cardiac muscle unit - "sarcomeres." The function of the sarcomere may be altered by an abnormality in the quality or quantity of a protein, which, in turn, affects normal myocardial contractility. The mechanism by which mutations of sarcomere-related genes cause heart hypertrophy remains unknown. However, structural abnormalities in HCM include abnormal myofibrils and irregular arrangements of cardiomyocytes, as well as coronary artery microvascular dysfunction. Mutations of MYH7, MYBPC3, TNNT2, TNNI3 (encoding sarcomeric proteins), LBD3 (encoding Z-disk proteins), and DES (encoding sarcomere-associated proteins) account for most cases of HCM with a known genetic etiology [4].

Several morphological forms exist as follows: asymmetric basal septal (classic HCM), concentric, reverse septal, neutral, and apical. Outside of Asian populations, apical HCM is less common; it occurs in 1-10% of non-Asians compared to 25% of Asians with HCM [2]. The syndrome is characterized by ventriculographic features, including a "spade-like" figure of the left ventricular cavity, among others [2]. It is more common in men than women, with an average age of presentation of 41.4±14.5 years. Associated findings include left atrial dilatation, increased left ventricular (LV) filling pressures, elevated cardiac biomarkers, apical aneurysm, and myocardial scar formation [2]. Also, present is small vessel disease resulting in myocardial ischemia from cavity obliteration, persistence of contraction in apical segments, regional perfusion defects, and chest pain [1,2].

Clinical manifestations of apical HCM include palpitations, dyspnea, fatigue, syncope, and sudden cardiac death, the latter related to apical infarction with aneurysm formation and malignant ventricular arrhythmia. Some patients will, however, be asymptomatic and diagnosed incidentally [1,2]. Some of these symptoms and many other features of the apical variant were clearly demonstrated in the patient presented.

The diagnosis of HCM is triggered by identifying a family history of HCM, symptoms such as chest pain, exertional dyspnea, syncope, and embolic events, and abnormal physical findings such as a systolic murmur and an abnormal 12-lead electrocardiograph. HCM is primarily an imaging diagnosis, as symptoms, physical findings, and ECG findings are non-specific. Based on guidelines, in adults, any telediastolic wall thickness ≥15 mm anywhere in the left ventricle, in the absence of abnormal loading conditions (cardiac or systemic) that could explain the hypertrophy, is diagnostic of hypertrophic cardiomyopathy [5]. Apical HCM is diagnosed by identifying LV hypertrophy predominantly confined to the LV apex, with an apical wall thickness ≥15 mm and an apical-to-posterior wall thickness ratio ≥1.5 [1]. Transthoracic echocardiography plays a crucial role in the diagnosis of HCM, due to its availability and ability to identify many of the structural and functional abnormalities associated with the condition. Echocardiography has limitations and pitfalls, including poor acoustic windows, inability to evaluate the LV apex in some patients, and foreshortening of the LV. Many of these shortcomings are resolved using cardiac magnetic resonance imaging [2]. In this report, an apical thickening of 15 mm was demonstrated on transthoracic echocardiography, although it could not be assessed with MRI.

Strain imaging using echocardiography or MRI is a valuable diagnostic tool, revealing regional strain defects (as indicated by peak systolic longitudinal strain) (Figure 2). The most emblematic of these is a polar map showing a "blueberry-on-top" pattern, which may serve as a surrogate for myocardial disarray at the apex in this patient [2]. Hughes et al. have described improved diagnostic criteria for apical HCM, based on a fixed threshold of >15 mm, which aligns with the classical diagnostic criteria but fails to account for many cases of apical HCM. A normal left ventricle typically tapers from the base towards the apex. Therefore, the threshold for hypertrophy at the apex should be <15 mm. Using cardiac MRI, they not only described "relative" apical HCM, characterized by typical electrocardiographic features, loss of apical tapering, and cavity obliteration, but also noted a maximum wall thickness <15 mm. This diagnosis was based on regional wall thickness values indexed for age, sex, and body surface area [6].

A review of the literature reveals a single case report of familial hypertrophic cardiomyopathy in Ghana [7]. Furthermore, a meta-analysis by Doku et al. did not directly identify HCM as a cause of heart disease in Ghana. However, the pooled prevalence of cardiomyopathies was 16.33 (range: 12.53-20.13), and that of idiopathic cardiomyopathies was 16.8 (range: 13.74-19.86). These two groups may include HCM [8]. Data on HCM from Africa are scarce [9,10]. We did not find case reports of apical HCM from Ghana or the West African subregion. At least two cases of apical HCM have been reported from the continent [11,12].

Management of HCM involves shared decision-making. Prevention of SCD must be discussed and implemented based on risk evaluation (HCM SCD risk score), with a patient with a significant risk of SCD receiving an implantable cardioverter-defibrillator (ICD) [5]. Those with symptomatic obstruction of the LV outflow tract may receive medical therapy, beta-blockers, verapamil, or disopyramide. A novel therapy, mavacamten, a small allosteric modulator of cardiac myosin and a strong negative inotrope, is now available in many developed countries for the management of HCM. If symptoms persist despite optimal medical treatment, the interventricular septum may be reduced via surgical myectomy or alcohol septal ablation, where available. Patients without left ventricular outflow tract obstruction (LVOTO) but with progressive heart failure despite optimal medical therapy may be placed on the heart transplant list, where available. HCM patients with atrial fibrillation, regardless of their CHA2DS2-VASc score, should receive anticoagulation, as this score is unreliable in the context of HCM [13]. Our patient was considered ineligible for ICD based on the previously described risk score.

Future management will be tailored to gene therapy, in which identifiable genes can be edited before they are expressed. A single-dose genetic therapy that can block pathological variants in mice has already been demonstrated [14]. An ongoing clinical trial is evaluating an adeno-associated virus-based delivery system that encodes a gene for myosin-binding protein C3 (MYBPC3) in individuals with HCM [14]. Other novel therapies include blebbistatin, which targets the troponin T mutation and decreases calcium^2+ ^sensitivity. Parvalbumin targets calcium^2+^ by decreasing its concentration. SERCA2a also targets calcium^2+^, but does so by increasing SERCA2 expression [4].

## Conclusions

Hypertrophic cardiomyopathy is the most common genetic cardiomyopathy. Its apical variant is more prevalent in Asian populations. A middle-aged Ghanaian male who has been experiencing chest pain for years presented with an ECG showing sustained ST depression and deep inverted T-waves. Two-dimensional echocardiography and strain analysis were suggestive of apical hypertrophic cardiomyopathy. Holter monitoring was unremarkable for malignant ventricular arrhythmia. The patient was treated with carvedilol 6.25 mg orally twice daily, resulting in the resolution of symptoms. This case highlights the diagnosis of the rare Yamaguchi syndrome in a West African patient.
